# “You treat your stress by doing what you’re supposed to do”: a qualitative inquiry into emotion regulation of paramedics and paramedic students in critical incidents

**DOI:** 10.1186/s12873-025-01228-6

**Published:** 2025-04-27

**Authors:** Branislav Uhrecký, Veronika Kučerová, Denisa Paksi

**Affiliations:** 1 Institute of Experimental Psychology, Centre of Social and Psychological Sciences, Slovak Academy of Sciences, Bratislava, Slovak Republic; 2 Institute of Applied Psychology, Faculty of Social and Economic Sciences, Commenius University in Bratislava, Bratislava, Slovak Republic

**Keywords:** Emergency medical service, Occupational stress, Paramedic students, Emotion regulation, Resilience

## Abstract

**Background:**

Emergency medical services (EMS) are among the professions with a high degree of responsibility and the frequency of critical situations. Existing research is largely quantitative and provides little insight into the specifics of critical incidents and the emotion regulation strategies used to manage them. Furthermore, little is known about the process by which an experienced paramedic is equipped with emotion regulation resources in the profession.

**Method:**

In this study, we interviewed 12 experienced paramedics (at least 4 years of practice) and 10 urgent medical care students about the most intense acute stressors they encounter and the emotion regulation that these stressors trigger.

**Results:**

Psychological distancing, attention management, cognitive framing and interpersonal self-regulation were used by both groups as means of emotion regulation. Identification with the professional role is a key aspect of maintaining a sense of psychological distance. A balance between distance and connectedness is sought. It is not so easy for paramedic trainees to achieve a sense of psychological distance from patients and relatives, and their attention may shift from the situation to themselves, leading to greater anxiety.

**Conclusion:**

Emotions and emotion regulation are taboo subjects in paramedic community, but greater awareness of them might be beneficial in psychological adaptation to work.

**Supplementary Information:**

The online version contains supplementary material available at 10.1186/s12873-025-01228-6.

## Introduction

When we think of emergency medicine, we typically think of extremely stressful emergency situations such as cardiopulmonary resuscitation, car accidents, traumatic injuries, anaphylactic shocks etc. Emergency situations with high level of responsibility, infant patients, or external threats are indeed considered as very stressful by paramedics themselves [1, 2]. Apart from situational acute stressors, paramedics are also burdened by chronic stressors such as shift work, lack of sleep, excessive duties, paperwork, demanding nature of patients, working with a substandard co-employee, conflicts with co-workers, interprofessional conflicts during emergency incidents or patient hospital admission [3, 4]. The confluence of acute and chronic sources of stress inspired a handful of studies about occupational stress and coping in emergency medical service (EMS) personnel.

Most of the research that has been done on the subject is concerned with the macro-level of coping, without reference to specific situations. Qualitative studies in this domain reveal that paramedics largely depend on detachment from emotions, specifically adopting a purely technical approach to work and decision making, and the use of black humour in informal communication with colleagues [2, 5–7]. Although emotionally-avoidant coping strategies are usually considered maladaptive in psychological literature [8], they could be effective for the specific context of EMS work. This notion is supported by a quantitative study by Mitmansgruber et al. [9], where more experiential avoidance and fewer meta-emotions (i.e., emotions to one’s own emotions) were related to greater mental well-being. Seeking social support, especially informal debriefing with colleagues after stressful incidents, is also mentioned among coping strategies [5, 6].

On the micro-level of acute stress, excessive amount is associated with the risk of human factor errors in short-term, and therefore, stress management is recognized as one of non-technical skills in EMS workers [10]. However, as they point out, there are not many studies about this particular skill, as compared to skills such as situation awareness or decision making. This is largely because non-technical skills are studied through observable behavioural markers, which is not possible in case of stress management. Nevertheless, a couple of research papers attempted to study it through retrospective verbal recall. When recalling critical incidents (i.e., exceptionally difficult or emotionally strong incidents) from their practice, paramedics speak of the necessity to find a balance between connection and detachment [11–13]. To achieve a sense of control, they disconnect from patients’ and their relatives’ humanity and lived experience, at least to some degree [12, 13]. While some paramedics prefer to be completely detached and therefore, they do not have to confront the dilemma between connection and detachment, others do acknowledge the value of emotions, caring, and kindness in their work [11]. Studies that utilize verbal recall immediately after the incident (specifically, a simulated task) further complement the picture of EMS workers’ coping arsenal [14, 15]. According to them, narrowing one’s attention on the task, having pre-established cognitive frames to process patient death and failure, and being in state of emotional distance, rather than a complete detachment, is a typical combination of emotion regulation mechanisms.

Emotion regulation (ER) and coping are largely overlapping, but also distinct concepts. Most notably, ER represents a more micro-level perspective, concerned with specific emotions and moment-to-moment micro-processes [16]. Both emotion regulation [17] and non-technical skills literature [18] suggest that ability to manage stress develops over time by biological ageing and gaining experience with stressful situations. Therefore, we can presume this ability in experienced EMS workers has evolved since they were paramedic students or junior paramedics. While empirical research on stress and stress management in paramedic students is sparse, what is available suggests that paramedic students are indeed prone to develop post-traumatic stress disorder and are not sufficiently mentally prepared for emotionally challenging incidents [19]. What kind of emotions regulation mechanisms develop in these formative years of their career is likely crucial for their adaptation to work.

Emotion regulation in paramedics and moreso in paramedic students is still insufficiently understood, yet is a critical skill for them to be able to undertake their role. Building one’s capacity to regulate emotions is an informal process that can be a hit or miss – some junior paramedics and paramedic students might find the way to adapt without any guidance, but others might not be so fortunate. For this reason, we find it worthwhile to look at both EMS seniors’ and juniors’ ER strategies, which is also the aim of our study. The results could be informative in formalizing and destigmatizing the process of emotional adaptation to EMS work.

## Methods

### Participants and recruitment

The data was collected from June 2020 to March 2022. The purpose of our sampling strategy was to include various levels of expertise and experience, and to allow comparisons between experienced and inexperienced paramedics. Such heterogeneity in the data set can also serve as a basis for deductions on how paramedic resilience is built over time. Consequently, the research sample consists of 12 paramedics (7 men, 5 women) and 10 paramedic students (6 men, 4 women). The age of senior EMS workers ranged from 25 to 51 years (AM = 34.27, SD = 8.62) and their experience with the job ranged from 4 to 20 years (AM = 10.36, SD = 5.66). Paramedic students’ age ranged from 21 to 38 years (AM = 25.30, SD = 7.02). All of them were third year students of urgent medical care and two of them had two years of experience with emergency situations due to voluntary practice during their first year, others had over one year of experience. Both urban and rural areas, and six out of eight Slovak provinces are represented in the research sample.

Recruitment of participants was realized through companies providing EMS in Slovakia and social networks. The HR departments agreed to send an e-mail containing information about the ongoing research project and contact information for the research team to their employees. Facebook groups related to paramedicine and individual profiles were also used to share the information about participant recruitment. A shared database of potential participants willing to take part in-depth interviews was created and utilized for multiple studies focusing on non-technical skills in paramedicine. The first seven EMS workers in our research sample were recruited from this databased and the remaining five were recruited via snowball method. This could be former classmates, former colleagues or friends working in EMS as we wanted to avoid having two participants from the same workplace. Paramedic students were contacted only through posts on specialised Facebook groups and specifically for the interviews about emotion regulation.

The vast majority of the interviews were collected in-person – two EMS workers and two paramedic students were interviewed online due to pandemic situation and subsequent government policies. The recruitment of further participants ended when interviews no longer brought new information on the studied subjects.

### Data collection

The data were collected via semi-structured in-depth interview (lasting from 50 to 150 min with paramedics, from 40 to 80 min with paramedic students) by the first and second author of the study. At the start of the interview, participants were asked about the kind of situations and incidents that cause stress in their work (or internship, in the case of students). Then we asked them to recall a few critical incidents from their practice which were challenging for their ability to handle their emotions. The interviewer went through two or three of them in greater detail. Afterwards, a more general inquiry into their emotion regulation strategies in paramedic profession followed (for a complete interview guide, see Supplementary information). Interviews were audio-recorded and transcribed word-for-word.

Participants were informed they were allowed to withdraw their participation at any time during the interview or even after completing it. It was also communicated by the interviewer that the content of the interview might elicit uncomfortable emotions – if that happened during or any time after the interview, they were encouraged to disclose it to the interviewer. All of this information including contacts on the research team was in the informed consent form which they signed. Participants were rewarded by vouchers in the value of 30€.

### Data analysis

The data analysis procedure for emotion regulation was inspired by template analysis, a specific form of thematic analysis which emphasizes hierarchy and structure in the coding process [20]. In template analysis, it is permitted to utilize a combination of theoretical and exploratory approach, meaning that coding template can be constructed a priori, but should be modified during data analysis to represent the data more accurately. This approach was also chosen by the authors of this study due to conceptually complex nature of emotion regulation. The initial coding template was constructed before the analysis based on major review papers of emotion regulation [21, 22] and previous works by the research team [14, 15], but the analysis was also open to novel categories and findings. The initial coding template presumed the presence of cognitive strategies (e.g., reappraisal, acceptance, self-distancing), attention management strategies (e.g., attentional narrowing, distraction), situation-focused strategies (e.g., problem-solving, interpersonal emotion regulation), and response focused strategies (e.g., expressive suppression). Each interview was analysed independently by a pair of coders who were primarily looking for affective states experienced by participants, their triggers, and strategies to regulate them. For affective states and their triggers, no a priori template was constructed, they were coded with a strictly exploratory approach. After analysing first six interviews, coding template was revised – triggers, affective states, and novel categories of emotion regulation were incorporated. During in-person meetings, coders went through each interview, compared their codes, and reached consensual agreement on final coding of the data. Subsequently, relationships between emerging categories were defined and higher-order categories were rearranged to correspond with subordinate categories. Thus, the higher-order categorisation of emotion regulation strategies in final template is different than in the a priori template because certain subcategories were not found in the data while others did or emerged as more elaborate and complex than initially presumed (e.g., psychological distancing as a separate category). The first author suggested the final list of categories and send them to co-authors for further discussion and approval.

## Results

The results are structured into two sections. The first section is dedicated to an overview of situations and aspects of the job that trigger heightened levels of physiological arousal, and it is followed by the section that presents strategies to regulate it.

### Emotionally adverse situations

Paramedics mentioned three types of incidents they consider extra-ordinarily stressful, namely – mass casualty incidents (such as car crashes), incidents involving children and young people, and incidents involving threat to the paramedic team. Mass casualty incidents were described as stressful due to the combination of their complexity (patient triage, inter-team cooperation, situation awareness) and time stress. Incidents involving children and young people are highly tense because the meaning of “saving life” is perceived as more palpable compared to situations involving elderly people, thus resulting in a greater anxiety and a sense of responsibility. These incidents can be difficult to process if they did not end well. A few critical incidents described by respondents involved a dispute with a healthcare co-worker (paramedic-driver, paramedic-physician, physician in hospital emergency admission) when a patient was in life-threatening condition.

*The younger the patient*,* the greater the emotional burden. At least for me.* (P12)

Incidents involving threat to paramedic team are arguably the most stressful since maintaining psychological distance or detachment from patients is often achievable for paramedics (see further in ER strategies), but a threat to oneself or a colleague is always existential and personal.

*Aggression*,* verbal threats – those we come across almost on daily basis. When we are resuscitating someone’s mother and we start to see there is no point in this*,* nobody gave her the first aid for an entire hour and they are expecting miracles from us … and a guy pulls a knife on us and says that if we don’t save her*,* there’ll be three dead bodies. Then you work under real stress.* (P5)

Paramedic students likewise consider mass casualty incidents and incidents involving children to be among the most stressful, if they had such an experience, but they also differentiate from senior paramedics in a number of ways. Their orientation in the equipment bag is not as swift and they are less technically skilled, which makes them worry they might be more of a burden than a help. Situation novelty is itself a major stressor, especially if it involves a serious, typically life-threatening condition of the patient. Uncertainty and missing information in briefing also make them anxious. And since their sense of self-worth in professional identity is not yet properly established, patronizing crew members can have a major negative emotional impact. In one instance, a student recalled a nervous physician who stressed her during the entire call, especially while she was trying to locate a suitable vein to secure intravenous access.

*He was standing there with the infusion and he was like ‘what’s the matter*,* what are we waiting for’. He must have seen I was trying to find the vein. I did not want to harm the patient by inserting the needle at the wrong spot since she was not in a critical condition. I was not used to standing there and looking over my shoulder*,* it was an immense pressure.* (P7 - student)

### Emotion regulation strategies

**Fig. 1:**
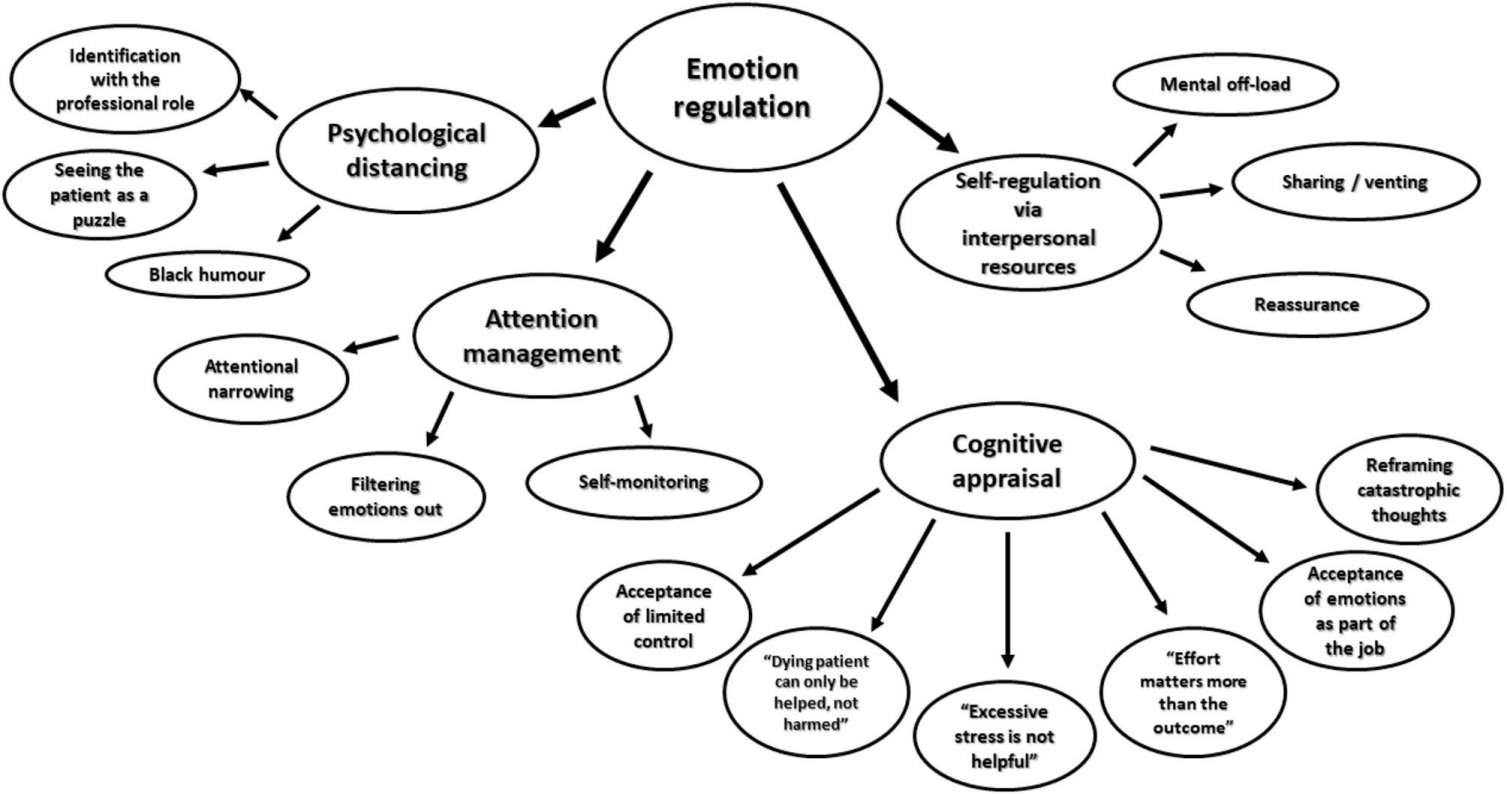
Identified emotion regulation strategies of paramedics and paramedic students

Four distinct categories of emotion regulation strategies were identified – psychological distancing, attention management, cognitive appraisal, and self-regulation via interpersonal resources (see Fig. 1). Below, we describe emotion regulation strategies in greater detail and present illustrative quotes.

#### Psychological distancing

The importance of detached and distanced approach to work was echoed throughout the words of our respondents on how a paramedic deals with emotionally negative experiences. The core characteristic of psychological distancing is minimizing the emotional aspect of a situation by making it less overwhelming and less personal. Our analysis revealed **identification with professional role** as the most important mechanism of achieving psychological distance. Due to this professional identity, all the suffering and drama that patients and their relatives are going through are seen through the lenses of a medical professional who puts boundaries in their personal engagement with emotions and stories of these people.

*The way I look at it* [dead bodies], *it’s my job. Maybe it would be different if it was my father … my relative … sure*,* I also see a person*,* but first and foremost*,* it’s my job and I have to do what I have to do … and not let it get too close to me or otherwise it gets over my head and then I’m done within two years.* (P3)

Being a professional was most typically understood as being the one who has the medical knowledge and technical skills, but also one who remains calm, focused and collected. While other qualities were also mentioned, such as being empathic, skilled in communication, or keeping up with the new guidelines, aforementioned features were universally agreed on

*You’re supposed to be the one who … who know something*, *who is gonna help them* [patients and relatives], *who is gonna solve the problem. Consequently*, *you have to adapt to it*, *you need to have faith in yourself*,*come there with a clear head if possible. Once you enter the room*, *you’re the one who must be responsible and decisive.* (P9)

Part of paramedic identity is being capable of taking care of your emotions on your own. Accordingly, experienced paramedics described the process of building resilience as a taboo topic in paramedic subculture. There is no formal training in stress management and psychological resilience, and in line with the ideal of a stoic paramedic, the subject is not spoken about in the open. Even if some find it difficult to cope with demands of the profession as students or juniors, “take it or leave it” approach was expressed by our respondents. Your either find means of coping on your own or you quit the job if you are unable to.

*I think everyone needs to cope* [with this job] *by themselves. Someone can adapt with easy*,* someone might take a long time. I had colleagues who could not get used to difficult emergencies for a long time. I remember a guy about whom we all thought he didn’t belong in the EMS*,* he was unable to cope with it. And now he is a top-notch paramedic. Somehow*,* he found a way* [of coping], *but we never discuss among ourselves how we build our protective shells.* (P6 - student)

In act of playing this role with its set of expectations, one’s subjective experience blends with it in experienced paramedics, but for paramedic students, identification with professional role is still in process and achieving balance between psychological distance and empathy is not as seamless. Some students felt too invested in patients’ suffering while others were radical in their attempts to achieve distance.

*You gotta stay professional*,* even when you know all is lost and there’s nothing you can do*,* but still remain a professional and act professional. In this job*,* you are a professional until you leave the patient. Then you can say anything*,* make jokes*,* curse. But on the scene*,* you stay professional from the start until the end. Sometimes it’s not possible*,* but you have to try.* (P3 - student)

Experienced paramedics recalled the role models that inspired them from their past days and in similar fashion, student paramedics spoke of observational learning and identification with the role – witnessing experienced professionals unmoved and cool-headed in difficult situations made them want to be like them. In some occasions, they would also receive direct messages and feedback accentuating the role of mastery and control over their emotional reactions.

*I told myself that if I want to do this job*,* I have to detach from it*,* I can’t let it affect me. I remember the specific moment I’ve learned this. It was during high school*,* my first internship after I’d turned eighteen. First time a patient died in front of my eyes. It struck me so much that I cried like a baby. I went to the changing rooms and the teacher said to me*:

*If you want to be a paramedic*,* you can’t let that affect you and you shouldn’t cry for this. It’s life*,* it has a beginning and an end. You can’t cry like that for every dead patient. And this is the moment I’ve learnt to be more cold and distant.* (P3 – student)

Along with assuming a professional identity, use of **black humour** was also mentioned as an effective way of becoming detached from the seriousness of a situation. This strategy was applied especially in the retrospect to defuse the tension of a past moment, resulting in seeing a bizzare scene rather than a drama when looking back. Another prime example of a detached perspective being helpful in dumping down intense emotions is when a young paramedic described how he was nervous in the ambulance during transport to a 4-year old child being resuscitated by parents, but the moment he arrived at the scene, suddenly a drama with a child’s life at stake **became a puzzle to be solved** and his feelings shifted. The rational and intellectual aspect of the job was at the forefront, which does not mean the emotional aspect ceased to exist, but was pushed to the background at least for the moment.

*I had such a storm in my head*,* that I couldn’t make any sense of it until I realized*,* that I’m at the address and have no idea what I’ve just been thinking about on the road there. But once you’re here on the scene*,* and start to ask the parents some questions and attend the patient*,* you start to put the pieces together and it’s like you’re solving some kind of a puzzle. And then we both became more relaxed.* (P9)

Most respondents acknowledged the importance of treating the patients as human beings, but the priority remains to do the job. Rather than a binary choice, it is a spectrum where a paramedic needs to find an ideal spot for him where he is as empathic as possible while protecting his mental well-being and capability to remain focused on the job.

#### Attention management

Managing one’s attention is a crucial part of non-technical skills such as situation awareness, decision making, and teamwork [18], but also for correct performance of technical aspects of the job. At the same time, attention management is a powerful tool of emotion regulation. Attention management means certain aspects of the situation are perceived at a given a moment and others are ignored, which determines how a situation impacts a person on an emotional level. For example, preoccupation of working memory can divert attention from the experience of negative emotion or negatively saturated stimuli [23]. Paramedics find it not just practical, but also emotionally comfortable to enter a bubble-like or tunnel-like state of **attentional narrowing**. For instance, one of the respondents described how he needed to “filter out” his emotions when he witnessed a dead body of a young girl after a car accident.

*I: Can you recall a specific moment about the situation that was most stressful for you?*

*R: Probably when the doctor said ‘there’s nothing you can do here’ when I wanted to help the girl. I understood that this is the end* [for her]. *In that moment*,* I needed to filter out my immediate emotional reaction. Somehow*,* I just blocked it inside my head … I stopped paying attention to it and shifted to another patient. And then*,* there was just the stress* [of providing care to other patients] *and other emotions were gone. I guess it was the adrenaline.* (P5)

In attention management domain, paramedics and paramedic students also **filter emotions out –** be it either emotional aspects of the situation or their own emotional reactions. At the same time, they admitted this process of preoccupation and blocking out the emotionally adverse stimuli is temporary and they became aware of it once the rush settles down, be it in the ambulance vehicle, at the station, or later after their shift. In some instances, they were made aware by their colleagues that emotional distress had been observable in their behavior such as hands shaking, loud and rapid speech, confusion etc.

*And the emotions*,* we become aware of them once we transport the patient. So*,* it’s not easy to tell you how we not get emotionally invested on the job. I guess it’s just because our brains are too busy with technical stuff*,* with the decision we make … and we do not quite have the time to realize what’s happening on an emotional level as the layman might*,* because he sees it differently.* (P6)

Keeping one’s focus on the job rather than the emotions it triggers or might trigger is largely supported by the motivation. Respondents often mentioned how they are primarily driven by their sense of duty or high purpose in critical situations, and therefore everything else takes a backseat. Along with their professional responsibility, there can also be an excitement about mission that is novel and challenging, which is consistent with findings on paramedics’ sensation-seeking [24]. Consequently, paramedics perceive physiological arousal as an impulse toward action and the best way to manage it is to perform.

*I do nothing about the stress*, *because this is what they taught me at school and I’m good at. They taught me that the adrenaline treats your stress. Yes*, *you are stressed! Your heart is pounding*, *your body enters a different mode. But I do nothing specific about the stress*, *the best thing to do is to do what’s your job. If you were so stressed that you freeze*, *that you just couldn’t do this job. So it’s only logical that you treat the stress by doing what you’re supposed to do. (I: So when you saw the baby and felt stressed*, *you were not affected by this*, *you did not freeze or anything*, *you just went on. Am I correct?) Exactly*, *exactly! No freezing*, *I prepared the ECG – remove the stickers*, *clip it on the patient and so on. You have the procedure that you have to do and you take care of the stress by doing it.* (P3 – student)

Most typically, being able to manage attention appropriately is not a completely separate mechanism, although functionally distinguishable, but one that co-occurs with psychological distancing. Being able to see the patient as a puzzle to be solved makes paramedics engaged and manage to focus and shift attention effectively and simultaneously, attentional preoccupation with the task fosters psychological distancing.

*You see a child on the table with no life signs*,* all blue. And you get this sad feeling. Sad*,* really sad. And the negative thoughts - ‘Oh my god*,* what happened here? Poor child.’ Such a sad feeling*,* and all the negative thoughts*,* but you tell yourself you gotta do it*,* you’re here to help. Even though you don’t feel like it*,* you have to perform.* (P8 – student)

Paramedic students only occasionally experienced a bubble or tunnel-like state on emergency calls. On the contrary, they were usually hypervigilant and they are unsure which aspects of the situation are worth paying attention to. In some instances, they can become **self-monitoring** toward their performance of manual work or physiological reactions of the body. They also tend to spend a lot of mental energy on anticipation of the emergency situation before and at the moment of arrival. As illustrated below, one of the students felt anxious about a call (middle-aged man after a fall off the ladder) since the notification until the patient was stabilized. His attention would divert to his bodily signals such as muscles tightening or hands shaking.

*For me*,* the notification was the trigger*,* because it was incomplete*,* lot of information was missing. I was new at the station and I didn’t know my way around the ambulance*,* so that was also stressful.* [At the scene, ] *I was shocked*,* like someone just slapped me. I felt the muscles tightening in my entire body. It was very uncomfortable and I was angry at myself for losing control.* (P1 – student)

Respondents also highlighted the role of standardized guidelines and automatization in routine mechanical tasks in attention management. Standardized procedures generally give paramedics’ a sense of certainty and self-assurance in their work performance, and support their state of flow. Besides, they simplify the job and bring ‘structure to chaos’, as some respondents echoed. Automatization of procedures also allows additional capacity for deliberation, communication with patients or relatives, or situation awareness, which consequently gives a greater sense of control over the situation.

*I really like the job on emergencies. When there’s something to do*,* there’s good equipment*,* then the thinking along with the physical activity … it’s like my hands do one thing and my mind thinks ahead*,* because there is not enough time. So the hands need to have the routine in them and then I’m enjoying it*,* when the manual work is almost doing itself.* (P11)

#### Cognitive appraisal

Both paramedics and paramedic students engage in meta-cognitive reflection of their attitudes, beliefs, and frames that are mostly related to professional responsibility and failure, which are emotionally significant also due to high stakes of the work. The process of cognitive appraisal determines the interpretation of a situation or evaluation of oneself, and thus modulates its emotional impact. Respondents often stressed the necessity to be **accepting of one’s error-proneness** and **limited control over outcomes on patient’s life**, and described several cognitive frames they employ to achieve this accepting attitude.

The necessity of accepting attitude stems from the uncertainty bounded in their working conditions, mainly limited options of patient diagnosis. Paramedics reflected that one can always find room for thoughts such as “could I have done more?”, because there is always something more you can think of when reflecting retrospectively on bad patient outcome with the knowledge one has now that he did not possess at the time of making decisions. Cognitive frames that help with being more accepting of less-than-ideal outcome of a call are not only directed at past events, but also toward possibility of future outcomes. An example of a helpful frame for situations with patients in critical conditions is one that states, **“if a patient is dying without my intervention**,** then I can’t do harm - I can only help him”**. One of the paramedics reflected on a situation where he had to decide between crew safety and CPR initiation in a following manner:

*First and foremost*,* I must think of my own safety. We have to return to the station and the one who does not breathe*,* he is not going to get worse. You can do no harm to him*,* he can only be helped.* (P3)

Most frequently expressed cognitive frame was one that **stressed effort rather than outcome** as a reason to feel guilty. In a life-or-death situation, the paramedic has no reason to feel guilty if he gave it his maximum effort to save the patient; and if that was not enough, then it simply was not meant to be. Therefore, an accepting attitude towards lack of omnipotent control and acceptance of death as a part of life is present in this cognitive framing.

*Sometimes you think if you had done more* [for the patient], *maybe he would have lived. But unfortunately*,* sometimes you can give it your all*,* have the best equipment*,* but I’m of the opinion that each one of us has a burning candle and when it’s supposed to burn out*,* it’s not in your powers to prevent it.* (P6)

While these frames do help paramedics’ conscience, it does not mean they are guilt-free as the outcome is not just determined by their effort, but also by the way they applied their medical knowledge to the situation. This is when the process of reconciliation with the past gets more complicated and they may get stuck in generating arguments for or against their procedure in the given time and space. Typically, it is not a lonely process and colleagues from or outside the paramedic crew are involved, as described by one paramedic on a situation of driving back to the station after unsuccessful CPR:

*In other situations*,* we usually make jokes*,* comment on what happened*,* but neither one of us felt like it this time. Just a few words summarizing how we’d proceeded. And there was this unvoiced question whether we had done things right*,* and we were sort of assuring ourselves that we had.* (P12)

With regard to the possibility of future failure and feelings of stress that are associated with it, paramedics tend to remind themselves that although some amount of stress is energizing, **overwhelming amount of stress is not a helpful reaction**. They report that being aware of this fact after years of service helps them in keeping the stress in check.

*You basically have two options*,* first one is that you get stressed and make chaos on the scene … and I know a couple of paramedics who say you gotta learn how to work in chaos. I don’t agree with that*,* I find it ridiculous. Why should I become part of the chaos? That’s not gonna help me or the patient. I’m of the opinion that if I come into chaos*,* I should try to sort things and bring order.* (P9)

While cognitive frames and psychological distance are helpful, but they do not make paramedics immune to its emotional impacts. Even though paramedics do not have personal relationships with patients and their relatives, with the exception of rural paramedics attending an incident involving their relatives or friends, they can still experience sadness and distress at the moment, but also years after when memories of them come back to mind. Paramedics refer to these pieces of their mind as ‘inner demons’ or ‘emotional baggage’ they have to carry for the rest of their lives. This is especially true if the memories are also burdened with guilt. Part of paramedic resilience to traumatic experience is **thus not struggling against ‘inner demons’**,** but accepting them** as a part of the profession they chose.

*In medical practice we have a saying that each doctor has his own cemetery and in the EMS*,* some say we all have demons haunting us. And those are the cases where we didn’t succeed*,* where we were too late*,* or when the patient didn’t receive the adequate treatment*,* or perhaps even received but didn’t make it nonetheless. And by not letting that person go out of our minds*,* he continues to live by our side. These are the exclamation marks in our head saying be careful so it doesn’t happen again.* (P11)

While similar cognitive frames were present among paramedic students, their position is different due to limited responsibility on duty, but that does not imply they do not feel stressed in critical situations. The presence of senior paramedics means they are not the ones who make decisions and bear formal responsibility, but it also means they are being watched and supervised by someone whom they often perceive as an authority. While paramedics typically have the luxury of already feeling established and confident in their role, paramedic students are merely aspiring for it. Consequently, failing or succeeding with assigned tasks is of importance to their self-esteem and relationships with their superiors. In case of perceived inadequacy, they also need to use their inner voice **to reframe “catastrophe” into something more acceptable**.

*My hands were sweating*,* but I tried to rationally calm myself by telling myself ‘I messed up*,* I could have done better*,* but I know it’ll pass’. Because I had a good rep on the station and they like me. I was worried a bit they might think I’m incompetent*,* but I was also telling myself that it was gonna be fine and it wasn’t such a big deal.* (P7 – student)

#### Self-regulation of emotions via interpersonal resources

While the interview focused mainly on intrapersonal level of emotion regulation, respondents also talked extensively about interpersonal aspects of emotion regulation. Some of them stressed emergency medicine is a team job and no paramedic operates alone. In this category, we included forms of emotion regulation that step outside the realm of intrapsychic process and their use depends on other people. We identified three regulatory functions of team and colleagues – sharing/venting, mental off-load, and reassurance.

To illustrate the importance of **sharing experiences** at work, we should first familiarise ourselves with the sometimes solitary nature of paramedic stress, especially in the context of close relationships outside of work. They do not feel completely free in sharing the experiences of their work days – what they encounter can feel too distant and even discomforting to their spouses. For this reason, they often believe in “what happens at work, stays at work” philosophy, with a few exceptions (for instance, two of our respondents were married to a paramedic). Colleagues are thus the exclusive social environment for venting and sharing their experiences.

*After emergencies like this*,* we usually have a conversation about what happened. Not like ‘oh my god*,* what have we done’*,* but more like ‘that was a fine call*,* finally something interesting’. Like*,* we share our joy. I hope that doesn’t sound like we’re happy about someone’s bad luck.* (P3)

Another vital regulatory function is a **mental off-load** in the sense that a partner with whom sufficient amount of sync has been achieved, who thinks alike and is reliable, enables the paramedic to put a portion of tasks out of his mind and focus solely on their portion of the work when on a call. This means less things to worry about and more comfort in the bubble-like state mentioned earlier. On the opposite side, the partner who is not reliable enough or frequent rotation of partners means additional burden on their mind in the form of having to check them or being more explicit in instructions.

*A lot of people forget that there’s a crew*,* not just you as an individual. That’s very important. A lot of them think like they’re alone and they must do everything by themselves. And that’s how they get stressed. But in my opinion*,* the crew works as a team. There’s two people having each other’s back and helping each other. And when the work is split*,* the stress goes away. Both take care of their own part.* (P3)

In the section dedicated to cognitive framing, we have mentioned that retrospectively reflecting performance and generating arguments for and against one’s procedure on an emergency call is not just an individual process, but involves conversations with colleagues to. While there is undeniably a rationalizing component to the process where colleagues participate as external feedback that is accounted in their reflections, we would also like to point to an emotional component. In the words of paramedics, reactions by colleagues can also act as a **reassurance** and an encouragement to accept there was nothing more they could have done, or to do better in future emergencies instead of blaming oneself.

*If I feel something heavy in my conscience*,* this is my best way of coping. I go to my colleague to get some feedback*,* it’s always a good conversation. Or if it was just a very stressful call*,* I always call her and hear how she would proceed in the situation.* (P8)

## Discussion

Through in-depth interviews and qualitative analysis, we have investigated which events or aspects of the paramedic profession are the most emotionally adverse in the eyes of paramedics and paramedic students, what strategies they use to regulate emotions on both an immediate and long-term basis, and how they understand resilience in their profession. With regards to emotionally adverse situations, both experienced paramedics and paramedic students agreed on mass casualty incidents, incidents involving children, and incidents with the presence of a physical threat as the most stressful, which is in line with previous findings [2–4]. In the case of senior paramedics, aggressive and non-cooperative patients, unindicated emergencies (i.e., patients whose medical problems are indicated for a visit at general practitioner rather than EMS call) or workload are mentioned as well, while for paramedic students, most life-threatening emergencies are considered stressful. Patronizing supervisors and anxiety regarding technical skills and orientation in equipment were also mentioned by them.

Although paramedic students do not bear responsibility for the outcome of emergency situations, they are still in the process of negotiation with their professional identity, which is getting stronger with years of study and practice [25], but can nonetheless be fragile. As professional identity is largely influenced by active learning and experiences in social environment [26], a negative feedback, especially when communicated in unsympathetic manner, can elicit intense negative emotions. Paramedic students and graduates have a desire to fit into the workplace culture [27] and for this reason, fear of failure under the supervision of esteemed seniors can be present despite lack of formal responsibility. Beside these considerations, paramedic students experience greater situation novelty and greater working memory preoccupation due to lack of internalized heuristics, automatisms and tacit knowledge [28], further contributing to their distress level.

On the side of the experienced paramedics, they acknowledged the seriousness and distress levels of critical emergencies, reported them as part of the job and the reason why they had signed up for this job in the first place. Respondents even spoke of them as exciting and infusing them with a sense of purpose and meaning. These findings are in line with literature on paramedic motivation, which reveals paramedics have higher-than-average sensation-seeking [24] and often chose their occupation to help those in urgent need [29].

Psychological distancing, i.e. looking at the patients as problems to be solved effectively rather than suffering human beings, was identified as a major mechanism of paramedic emotion regulation. Most paramedics and students were able to consciously reflect on this process and described it as a necessity to carry out their job, at least to some degree. While few of the respondents advocated for absolute detachment from the patients and their relatives, most of them embraced the idea of finding a balance between distance and relatedness that is an essential component of healthcare work [30]. The use of emotional avoidance in paramedic work is not novel [5, 6, 12, 15] and while viewed negatively by some authors [7], it can be beneficial for work performance [9]. Our findings further contribute to the literature by expanding on the role of professional identity in formation and employment of distancing strategies.

Distancing is supported by attention management strategies that help in filtering out the distracting and emotional aspects of the situation – paramedics often referred to it as a bubble-like or tunnel-like state, which is an identical finding to a previous study by the authors on paramedics’ emotion regulation in simulated tasks [14, 15]. Preoccupation of working memory is thus achieved, which diverts attention from the experience of negative emotion or negatively saturated stimuli [23]. Many respondents reported experiencing little to no emotional reactions during emergency situations, but this changes once they hand the patient over at the emergency hospital admission. However, a study by Peifer et al. [31] suggests a different interpretation. As in many other studies, paramedics reported low levels of stress, but physiological markers painted a whole different picture. Thus, paramedics may just be unaware of their affective states. Nevertheless, experimental studies suggest that attention-based ER strategies are effective, especially when the emotional stimuli are intense [32].

For paramedics, death and suffering are not just concepts in their rational mind, they are a visceral reality they regularly experience and therefore, they do not have the luxury of ignorance toward them. They have to find ways to accept their presence and to deal with the responsibility over someone’s life and health. To do so, they employ several cognitive frames, but one in particular – “if I did everything in my powers and knowledge for the patient, I need not to feel guilty, whatever the outcome”. However, the causality of the outcome for patient is always a grey zone. If the paramedic did not have the sufficient knowledge, could he not do something to have better knowledge or to acquire the information from the patient more efficiently? While guidelines offer sufficient sense of safety in most cases, this is not always the case, which is a moment when paramedics mostly engage in a process of discussions with their colleagues and superiors.

While we put our focus on intra-personal emotion regulation, it was impossible to omit strategies relying on other people, mostly colleagues, as they were mentioned spontaneously by our respondents as a vital component of their coping repertoire. However, we did not include strategies aimed at regulating others’ emotions, only the ones that paramedics and paramedic students used to regulate their emotions via other person. Experience sharing and venting, which was commonly mentioned by paramedics in our research sample, are well-known means of emotion regulation and coping in emergency services [33]. In case of paramedics, this can go hand-to-hand with psychological distancing as stories they share and the way they reflect on them is often coated in black humour [34]. Paramedics also mentioned how presence of a reliable partner helps them off-load their mind and narrow their attention, which is typically their comfort zone during emergencies. Colleagues can also offer reassurance of their procedure when they retrospectively experience doubt over its appropriateness. For future research, we believe greater focus on interpersonal emotion regulation, especially toward crew partners, patients and their relatives, would be beneficial.

Interviews with paramedic students reveal that they try to use similar emotion regulation strategies, but do not yet have the sufficient mental resources to use them as effectively. They try to acquire and maintain a sense of psychological distance through identification with professional role, but they do not feel as inhabited and self-assured in this role with regards to their knowledge, technical skills, or ability to manage emotions [27]. On the contrary, they sometimes tend to self-monitor their performance and bodily physiology when stressed. It is well-established that self-focused attention can further exacerbate anxiety [35]. They also do not have automatism, heuristics, and intuitions that enable attentional narrowing. For students, being overwhelmed by stimuli is a common experience as they do not know which aspects of the situation are relevant and which can be filtered out. Cognitive framing is present, but is used slightly differently due to the different position of students. Paramedics aim to accept negative patient outcome, which is possible if it is not perceived as failure to follow guidelines and lege artis procedures, and perceived failure generates guilt and regret. Paramedic students do not bear responsibility, but they look for positive feedback or want to avoid criticism from supervisors. The aim of cognitive framing is thus to accept perceived failure as a natural part of the learning process.

As far as limitations of our study are concerned, the sampling bias might have a great influence on results in such a delicate and, to some degree, even taboo topic. We can presume that paramedics and students with more repressive and denialistic coping style were less likely to participate in our research and thus, our results might not fully represent studied phenomena in its full scale. There is also a possibility of cultural specifics of EMS work in Slovak settings, which complicates generalisability of findings. Nevertheless, most findings are in line with previous literature and elaborate them in further detail.

## Conclusions

Paramedics and paramedic students find critical incidents with high stakes emotionally difficult, but trained paramedics already have an established repertoire of emotion regulation strategies. Psychological distancing, attention narrowing, cognitive frames, and interpersonal resources (crew mates, colleagues) were among the major mechanisms of emotion regulation. Paramedics students are largely aware of these mechanisms, but are still in the process of internalizing them. Achieving a balance between relatedness and distance toward patients and their relatives can be a struggle. Their attention management is not supported as firmly by self-confidence, automatisms and heuristics, which results in switching attention from the situation to themselves. According to both paramedics and students, becoming psychologically resilient to critical incidents in their occupation is an individual process that is not openly talked about, which is in line with previous studies reporting on the masculine culture in EMS [7, 34]. We suggest that introducing more open discourse on emotion regulation in paramedic culture, and normalization and formalization of emotional adaptation to the work in EMS, can be helpful to present-day and future paramedics.

## Electronic supplementary material

Below is the link to the electronic supplementary material.

Supplementary Material 1

## Data Availability

The datasets generated and analysed during the current study are not publicly available since this would compromise the informed consent signed by the participants. The informed consent contains a statement that only research project members would have access to the raw data and that findings would be presented in an anonymised way.
