# Supplementary material for: “You treat your stress by doing what you’re supposed to do”: a qualitative inquiry into emotion regulation of paramedics and paramedic students in critical incidents
Source: BMC Emerg Med. 2025 Apr 27;25:73. doi: 10.1186/s12873-025-01228-6 (PMC12036201; doi:10.1186/s12873-025-01228-6)
Supplement: Supplementary file 1 — Supplementary Material 1 [file 12873_2025_1228_MOESM1_ESM.docx]

**Supplementary information**
*Interview guide*

| **Basic information about participant** |
| --- |
| Gender |
| Age |
| Number of years in EMS |
| Number of years in healthcare |
| Do you work in one stable crew or do you rotate? |
| **Stressors and critical incidents** |
| What kind of situations in your work make you feel most stressed and anxious? |
| Try to recall some of the most emotionally difficult emergencies you experienced. Describe each one of them shortly. |
| Pick one that stands out. We will have time to go through another one or two during the interview. |

**BEFORE ARRIVAL**

| **Initial information** |
| --- |
| What was the information from the operation centre? |
| What was your physical and mental state before the call? |
| **Preparation** |
| What was your reaction? Did you have any expectations and hypothesis? (If you did, specify) |
| How did you feel about the call? Did you feel stressed? |
| (If he/she felt stressed: Did you do anything to alleviate your tension?) |

**ON THE SCENE**

| **Situation** |
| --- |
| What was the scene like? What did you notice first?  What was your evaluation of the situation? |
| **Emotions and emotion regulation** |
| What was it like for you emotionally?  What was it specifically that elicited that emotion? |
| What was going through your head at that moment? |
| What was your course of action?  What was the intention behind your action?  Did you notice any influence of your emotions on your behavior? |
| **Continuation** |
| What happened next? (environment, patient status, actions of colleagues and other actors)  What was it like for you? Did anything change about how you were feeling? |

*The questions go in a loop with each emotionally significant change in situation or their processing of the situation*.

**AFTER THE EMERGENCY CALL**

| **Immediately after** |
| --- |
| What were your feelings after the emergency? |
| Did you process what happened in any way? How? |
| **The most difficult moment / aspect** |
| What was emotionally the most difficult moment or aspect of this emergency call? |
| Did you notice how it affected your bodily reactions and information processing? Please, specify. |
| How did you regulate the emotion it elicited in you? |
| Can you think of other ways a paramedic might regulate his emotions in this type of situation? |
| **Reflection** |
| Do you ever go back to the situation? What are your thoughts?  Would you change anything about the way you handled it?  If you imagine a paramedic who is not skilled in handling his emotions, how do you think it could go? |

*The questions repeat when going through another critical incident*

**GENERAL INQUIRY INTO EMOTION REGULATION**

| **Motivational factors** |
| --- |
| What motivates you in this job?  What role do you think it plays in your ability to regulate emotions? |
| **Mental resilience** |
| Can you tell me generally about the mechanisms you rely on when handling your emotions?  What are your external resources for coping with occupational stress? (e.g. colleagues, peer groups, family)?  How does a paramedic become resilient to stress?  What are your emotion regulation / coping strategies outside the work setting? Are they the same or related to those you use on the job? |
| Do you discuss emotions and the way you handle them with your colleagues? |
